# Identification of a Novel Fungus, *Leptosphaerulina chartarum* SJTU59 and Characterization of Its Xylanolytic Enzymes

**DOI:** 10.1371/journal.pone.0073729

**Published:** 2013-09-09

**Authors:** Qiong Wu, Yaqian Li, Yingying Li, Shigang Gao, Meng Wang, Tailong Zhang, Jie Chen

**Affiliations:** School of Agriculture and Biology, Shanghai Jiao Tong University, Shanghai, China; Russian Academy of Sciences, Institute for Biological Instrumentation, Russian Federation

## Abstract

Xylanolytic enzymes are widely used in processing industries, e.g., pulp and paper, food, livestock feeds, and textile. Furthermore, certain xylanotic enzymes have demonstrated the capability to improve the resistance and immunity of plants. Screening of high-yield microbial xylanolytic enzyme producers is significant for improving large-scale cost-effective xylanolytic enzyme production. This study provided new evidence of high-level xylanolytic enzyme production by a novel fungus, designated *Leptosphaerulina chartarum* SJTU59. Under laboratory conditions, *L. chartarum* SJTU59 produced xylanolytic enzymes of up to 17.566 U/mL (i.e., 878.307 U/g substrate). The enzyme solution was relatively stable over a wide range of pH (pH 3.0 to pH 9.0) and temperature (40°C to 65°C) while showing high resistance to the majority of metal ions tested. Composition analysis of the hydrolytic products of xylan showed sufficient degradation by xylanolytic enzymes from *L. chartarum* SJTU59, mainly the monosaccharide xylose, and a small amount of xylobiose were enzymatically produced; whereas in the presence of sufficient xylan substrates, mainly xylooligosaccharides, an emerging prebiotic used in food industry, were produced. In addition, the xylanolytic enzyme preparation from *L. chartarum* SJTU59 could initiate tissue necrosis and oxidative burst in tobacco leaves, which may be related to enhanced plant defense to adversity and disease. *L. chartarum* SJTU59 possessed a complex xylanolytic enzyme system, from which two novel endo-β-1,4-xylanases of the glycoside hydrolase (GH) family 10, one novel endo-β-1,4-xylanase of the GH family 11, and one novel β-xylosidase of the GH family 43 were obtained via rapid amplification of complementary DNA ends. Given the high yield and stable properties of xylanolytic enzymes produced by *L. chartarum* SJTU59, future studies will be conducted to characterize the properties of individual xylanolytic enzymes from *L. chartarum* SJTU59. xylanolytic enzymes-encoding gene(s) of potential use for industrial and agricultural applications will be screened to construct genetically engineered strains.

## Introduction

Xylanolytic enzymes are an extensive group of enzymes (EC 3.2.1.x) [1]. An abundance of diverse xylanolytic enzymes are currently known to catalyze the hydrolysis of xylan. These xylanolytic enzymes are generally classified using standard means based on primary structure comparisons of catalytic domains and enzymes in families of related sequences [2]. At least a total of 131 glycoside hydrolase (GH) families exist in the carbohydrate-active enzyme (CAZy) classification system [3]. However, only members of GH families 5, 7, 8, 10, 11, and 43 contain truly distinct catalytic domains with a demonstrated xylanolytic enzyme activity. Among these, endo-β-1,4-xylanases (EC 3.2.1.8) are normally found in GH families 10 and 11, whereas β-xylosidases (EC 3.2.1.37) in the GH family 43. By contrast, xylanolytic enzymes belonging to GH families 5, 7, and 8 have been studied to a lesser extent. Endo-β-1,4-xylanase, which catalyzes the hydrolysis of β-1,4-xylosidic linkages of xylan backbone, and β-xylosidase, which catalyzes the hydrolysis of nonreducing end-xylose residues from xylooligosaccharides, both belong to the group of xylanolytic enzymes [1].

Applications of xylanolytic enzymes can be found in feed, food, pulp/paper, textile, deinking, metal-polluted sewage treatment and so on. In grain processing for feeds or energy sources, xylanases and β-xylosidase are commonly used in solid-state fermentation of agro-industrial wastes such as rice straw [4], wheat bran [5], and sugarcane bagasse [6]. As for the food industry, xylanases are beneficial enzymes in hydrolysis of xylan and production of xylooligosaccharides, an emerging prebiotic beneficial for human intestinal health [7]. In bleaching and pulp/paper industries, xylanase pretreatment is considered efficient for improving the product quality [8]. For plant disease management, the xylanase II gene has been shown to induce ethylene biosynthesis, which potentially improves the resistance and immunity of host plants to specific diseases [9].

Isolation and characterization of high-efficiency xylanolytic enzymes have become an important focus of research for decades, given their excellent industrial and agricultural values [7], [9]. To achieve high-yield, cost-effective production of xylanolytic enzymes, substantial efforts have been continuously made to screen for microbial xylanolytic enzyme producers from soil [10], composting materials [11], and agricultural and industrial wastes [12], [13]. Representative microbial xylanolytic enzymes producers include *Trichoderma*, *Aspergillum*, and *Penicillium* (fungi) [1], [14]–[16], as well as *Streptomycetes* and *Bacilli* (bacteria) [17], [18]. Enhanced production of xylanolytic enzymes has been achieved in *Trichoderma longibrachiatum* (about 600 U/g substrate) [19]. *Aspergillus fumigatus* (219.5 U/g substrate) [15], *Penicillium janthinellum* (55.3 U/mL) [20], and *Bacillus subtilis* (12 U/mL) [21] under optimized growth conditions.

In the present study, we demonstrated high-level xylanolytic enzymes production by a novel fungal strain of *Leptosphaerulina chartarum* SJTU59. Physicochemical properties of xylanolytic enzymes from the novel fungus were examined over a wide range of temperature (10°C to 100°C) and pH (pH 3.0 to pH 11.0) under laboratory conditions. In addition, we evaluated the resistance of the enzymes to a variety of metal ions and chemical compounds under laboratory conditions. Then, the hydrolytic products of xylan degraded by xylanolytic enzymes from *L. chartarum* SJTU59 were analyzed via thin-layer chromatography (TLC). Furthermore, tissue necrosis was initiated in tobacco leaves injected with xylanolytic enzyme preparation from *L. chartarum* SJTU59. Oxidative burst, which is related to enhanced plant defense against adversity and disease, was also detected in necrotic lesions via 3, 3′-diaminobenzidine (DAB) staining.

For industrial and agricultural uses, we performed a detailed study of *L. chartarum* SJTU59 xylanolytic enzymes, from which four full-length coding sequences (CDS) of xylanolytic enzymes were obtained via rapid amplification of complementary DNA (cDNA) ends (RACE). Sequence alignments and phylogenetic analyses showed that the four novel genes encode two endo-β-1,4-xylanases of the glycoside hydrolase (GH) family 10, one endo-β-1,4-xylanase of the GH family 11, and one β-xylosidase of the GH family 43. Results provided new understanding of the function of *L. chartarum* SJTU59 in xylan-degrading processes as well as valuable data for potential use in relevant industrial and agricultural applications. Xylanolytic-enzyme gene(s) for potential industrial and agricultural applications will be screened to construct genetically engineered strains.

## Materials and Methods

### Fungal Cultivation and Isolation

The novel fungal strain, designated *L. chartarum* SJTU59, was isolated from a contaminated broth in the Department of Resource and Environmental Science, School of Agriculture and Biology, Shanghai Jiao Tong University, Shanghai, China. In another experiment involving enzyme activity assay of a transgenic *Streptomyces* strain with the xylanase gene, a semisynthetic xylanolytic enzyme-inductive fermentation broth (2% corncob meal, 0.4% NH_4_NO_3_, 0.05% K_2_HPO_4_, 0.05% MgSO_4_⋅7H_2_O, 0.05% KCl, and 0.001% FeSO_4_⋅7H_2_O) was contaminated by *L. chartarum* SJTU59, which produced large amounts of xylanolytic enzymes. Then, *L. chartarum* SJTU59 mycelia were harvested, washed in distilled water, and cultured on a potato-dextrose agar (PDA) plate in a thermostatic incubator (25°C, 1 d). Afterward, one of the outermost and youngest mycelia was harvested and cultured on a PDA plate in a thermostatic incubator (25°C, 7 d) for morphological analyses.

### Morphological, Molecular, and Phylogenetic Analyses with Internal Transcribed Spacer (ITS) and Elongation Factor 1α (EF-1α) Sequences

For morphological observation, mycelia with spores of *L. chartarum* SJTU59 were harvested with an inoculating loop and examined under a DM 2500 M microscope (Leica, Germany) at 400×magnification.

For molecular analysis, *L. chartarum* SJTU59 was grown in a potato-dextrose (PD) broth on a 180 rpm rotary shaker (28°C, 3 d). After the removal of supernatant with multiple layers of gauze, total genomic DNA (gDNA) was extracted from the precipitate using hexadecyl trimethyl ammonium bromide (CTAB) method [22]. The gDNA extract was electrophoresed on 1% agarose gel and subsequently served as the template for PCR amplification of ITS and EF-1α sequences with specific primer sets, ITS1-F/ITS4 [23] and EF1-728F/EF1-968R [24] (Table 1). For ITS and EF-1α amplification, the 25 µL PCR reaction contained 9.5 µL of ddH_2_O, 12.5 µL of premix Taq™ (Takara, Japan), 1 µL of primer-F (ITS1-F or EF1-728F, 10 µM), 1 µL of primer-R (ITS4 or EF1-968R, 10 µM), and 1 µL of gDNA. PCR amplification was conducted on a thermal cycler (Eppendorf, Germany) under the following conditions: pre-heating at 95°C for 5 min, followed by 33 cycles of denaturation at 94°C for 1 min, annealing at 55°C for 1 min, and extension at 72°C for 1 min, and final extension at 72°C for 10 min. PCR products were electrophoresed on 1% agarose gel, stained with ethidium bromide dyestuff D1210 (Applygen, China), and then ligated into the pMD18-T Easy vector (Takara, Japan) for DNA sequencing (Biosune, China).

**Table 1:** Primers used in this study.

Primer name	Primer sequence (5′→3′)
ITS1-F	CTTGGTCATTTAGAGGAAGTAA
ITS4	TCCTCCGCTTATTGATATGC
EF1-728F	CATCGAGAAGTTCGAGAAGG
EF1-968R	TACTTGAAGGAACCCTTACC
X10-F	CTACGACTGGGAYGTNIBSAAYGA
X10-R	GTGACTCTGGAWRCCIABNCCRT
X11-F	AACTGCTACCTGKCNITNTAYGGNTGG
X11-R	CCGCACGGACCAGTAYTGNKIRAANGT
XylDF	GCAAGCAGCTGTGGGCNCCNGAYGC
XylDR	ACTCCACGATGCTGTGGTGNGTNGTCCANCC
*xyn-l1*-F	GGACCGGTCAATTGAGTACCTCA
*xyn-l1*-R	CTACCTACGCGTACGACCTCAGT
*xyn-l2*-F	TGACCAGCTCTCGATCATAC
*xyn-l2*-R	CACCAACCAAGGTGTTGTAC
*xyn-l4*-F	CTTCTTGCTGCGGCAGCTG
*xyn-l4*-R	GCGGTCTCTCAAGTCTTTC
*xys-l5*-F	TCGCATATTTCTCTTCGAAC
*xys-l5*-R	GCCCACGACTTATTGTTTTA

The underlined letter represents degenerate area: R = A/G, Y = C/T, M = A/C, K = G/T, S = C/G, W = A/T, H = A/T/C, B = C/G/T, V = G/A/C, D = G/A/T, N = A/T/G/C; and I = hypoxanthine.

The obtained ITS and EF-1α sequences of *L. chartarum* SJTU59 were compared with the GenBank database using the NCBI Web BLAST service (http://www.ncbi.nlm.nih.gov/BLAST/). Nucleotide alignments were done via DNAStar software.

For phylogenetic tree construction of nrDNA ITS (incl. ITS1, 5.8S and ITS2), sequences were downloaded from the GenBank database and then, together with the sequence of *L. chartarum* SJTU59, 13 representative sequences of known strains (incl. 16S rDNA sequences of *Streptomyces* and *Bacillus*, two representative bacteria with high production of xylanolytic enzymes), were imported into the MEGA 4.0 software [25]. For phylogenetic tree construction of EF-1α gene, sequences of the closely related species and outgroup were downloaded from the related literature [24] and then, together with the sequence of *L. chartarum* SJTU59, 12 representative sequences of known strains, were imported into the MEGA 4.0 software [25]. Multiple sequence alignment was performed using the Clustal W method and phylogenetic trees were re-constructed using the neighbor-joining method. Confidence for tree topology was estimated based on 1,000 bootstrap replicates.

### Xylanolytic Enzyme Activity Assay

The productivity of xylanolytic enzyme by *L. chartarum* SJTU59 was tested with two broth media, i.e., the PD seed broth and the semisynthetic xylanolytic enzyme -inductive fermentation broth (2% carbon source, 0.4% NH_4_NO_3_, 0.05% K_2_HPO_4_, 0.05% MgSO_4_⋅7H_2_O, 0.05% KCl, and 0.001% FeSO_4_⋅7H_2_O). The latter was prepared with agricultural waste as carbon source, e.g., 80-mesh sieve powders of corncob meal, wheat bran, sugarcane bagasse, wood chips, or cottonseed hulls.

Mycelia of *L. chartarum* SJTU59 were inoculated into the seed broth at 1∶500 (w/v) and then incubated on a 180 rpm rotary shaker at 28°C for 3 d. Then, the precipitate was collected by filtration with multiple layers of gauze. One gram of the precipitated seed was added to 50 mL of fermentation broth and then incubated on a 180 rpm rotary shaker at 28°C for 4 d. Xylanolytic enzyme activity of the fermentation supernatant was assayed by determination of enzymatically produced reducing-sugar content (i.e., xylose) using the 3,5-dinitrosalicylicacid (DNS) method [26]. The 2 mL reaction mixture contained 1.8 mL of 1% beechwood xylan (Serva, Germany) in 0.1 M sodium acetate buffer (pH 5.0) and 0.2 mL of diluted crude enzyme extract (30 µL crude enzyme extract in 170 µL of sodium acetate buffer). After gentle vortexing, the mixture was incubated in a 50°C water bath for 10 min. Reaction was terminated by adding 3 mL of DNS reagent and heating at 100°C for 10 min. Prior to enzyme activity assay, the mixture was diluted thrice with 0.1 M sodium acetate buffer (pH 5.0). Absorption of the appropriately diluted sample was measured at 540 nm (A_540_) using a UV-1800 spectrophotometer (Mapada, China). Uninoculated fermentation broth and xylose were used as blank control and standard, respectively [26]. Each measurement was performed in triplicate. One unit (U) of xylanolytic enzyme activity was defined as the amount of enzyme required to release 1 µmol xylose per minute under the standard conditions described above. The values were displayed with two kinds of units (U/mL and U/g substrate) to compare with existing data. Enzyme activity was statistically analyzed via SPSS software and the graph was prepared with Origin 8.0 software.

### Evaluation of the Effects of pH, Temperature, and Chemicals on Xylanolytic Enzyme Activity

Optimum pH for xylanolytic enzyme activity from *L. chartarum* SJTU59 was determined at 50°C in three buffer systems containing 1% beechwood xylan, i.e., McIlvaine buffer (pH 3.0 to pH 7.0), Tris-HCl (pH 7.0 to pH 9.0), and 0.1 M glycine-NaOH (pH 9.0 to pH 11.0) [27]. Stability of xylanolytic enzymes at different pH levels was evaluated by residual enzyme assay under standard conditions (pH 5.0, 50°C, 10 min) after pre-incubation without substrate in the above mentioned buffer solutions (pH 3.0 to pH 11.0, 4°C, 12 h).

Optimum temperature for xylanolytic enzyme activity was determined over the range of 10°C to 100°C in 0.1 M sodium acetate buffer (pH 5.0) containing 1% beechwood xylan. Thermostability of xylanolytic enzymes was determined by residual activity assay under standard conditions (pH 5.0, 50°C, 10 min) after pre-incubation without substrate in 0.1 M sodium acetate buffer (pH 5.0) at 40, 50, or 60°C for various periods [27], [28].

To determine the effects of metal ions, chemical modifiers and detergent on xylanolytic enzyme activity, 1 or 10 mM of Li^+^, Na^+^, K^+^, Ca^2+^, Mg^2+^, Cu^2+^, Zn^2+^, Ni^2+^, Fe^3+^, Cr^3+^, Co^2+^, Al^3+^, Ba^2+^, ethylene diamine tetracetic acid (EDTA), or sodium dodecyl sulfonate (SDS) was added to the enzyme solution, respectively. After gentle vortexing, residual xylanolytic enzyme activity was assayed under standard conditions (pH 5.0, 50°C, 10 min). Control experiments were carried out in the absence of each compound under the same conditions [27], [28]. Each measurement was performed in triplicate. Enzyme activity was statistically analyzed via SPSS software and the graph was prepared with Origin 8.0 software.

### Composition Analysis of Hydrolytic Products

The composition of hydrolytic products of beechwood xylan by xylanolytic enzymes from *L. chartarum* SJTU59 was analyzed on silica gel GF254 plates (Xinchu, China) by TLC. The 1 mL reaction mixtures were prepared with an increasing volume of crude enzyme extract and a decreasing volume of 1% beechwood xylan (i.e., 20 µL +980 µL; 30 µL +970 µL; 50 µL +950 µL; 200 µL +800 µL; 300 µL +700 µL; 500 µL +500 µL; and 800 µL +200 µL) in 0.1 M sodium acetate buffer (pH 5.0). The mixtures were incubated at 50°C for 24 h. Then, 25 µL aliquots of the hydrolytic products were spotted onto TLC plates. The plates were developed with butanol-acetic acid-water (2∶1:1, v/v), sprayed with a staining solution of (1% (w/v) diphenylamine and 1% (v/v) aniline in acetone, and then heated at 120°C in an oven for 10 min [27]. A mixture of xylose and xylooligosaccharides (X_1_–X_4_, 5 mg/mL each) was used as standard.

### Plant Inoculations and Assays of Tissue Necrosis and Oxidative Burst

Tobacco (*Nicotiana benthamiana*) plants were grown to the flowering phase in soil under 10 h light/14 h darkness in a 24°C phytotron. A total of 30 µL of filtered xylanolytic enzyme preparation from *L. chartarum* SJTU59, blank broth (control), and distilled water (control) were injected into tobacco leaves using 1 ml syringes, respectively. Injected leaves in each group were of the similar size and position in the same tobacco plant. The inoculated plant was placed in darkness in a 24°C phytotron, and tissue necrosis was observed every 12 h. For oxidative burst assay, injected leaves were stained in DAB solution (pH 3.8) for 8 h in darkness, boiled in a solution of ethanol, glycerin and acetic acid (1∶1:1, v/v) for 10 min to 15 min, and washed twice with 60% ethanol [29]. Each measurement was performed in triplicate.

### Conserved Region Amplification of Novel Xylanolytic Enzyme Genes and Sequence Analyis

Conserved regions of novel xylanase and xylosidase genes of GH families 10, 11, and 43 were PCR-amplified using three sets of degenerate primers (Table 1), namely, X10-F/X10-R [30], X11-F/X11-R [30], and XylDF/XylDR [31], respectively. The 25 µL PCR reactions contained 7.5 µL of ddH_2_O, 12.5 µL of premix Taq™ (Takara, Japan), 1 µL of primer-F (10 µM), 1 µL of primer-R (10 µM), and 3 µL of gDNA. The PCR conditions were as follows: pre-heating at 95°C for 10 min, followed by 35 cycles of denaturation at 95°C for 1 min, annealing at 50°C for GH 10 (52°C for GH 11 and 55°C for GH 43) for 1 min, and extension at 72°C for 1 min, and final extension at 72°C for 10 min. PCR products were electrophoresed on 1% agarose gel and visualized by staining with ethidium bromide dyestuff D1210 (Applygen, China). Visible bands of interests were excised. DNA samples were purified and ligated into the pMD18-T Easy vector (Takara, Japan) for DNA sequencing (Biosune, China). Conserved genomic DNA regions of xylanase and xylosidase genes were analyzed and identified using the NCBI Web BLAST service (tblastx; http://www.ncbi.nlm.nih.gov/BLAST/). Introns were predicted using the ExPASy Web service (http://web.expasy.org/translate/) and the GT-AG rule.

### Full-length Amplification of Novel Xylanolytic Enzyme Genes and Sequence Analysis

Mycelia of *L. chartarum* SJTU59, cultured in semisynthetic xylanase-inductive fermentation broth on a 180 rpm rotary shaker for 2 d, were collected by filtration with multiple layers of gauze and then washed twice with distilled water.

Total RNA was prepared using a TRIzol kit (Tiangen, China). Total RNA was reverse-transcribed using SuperScript trademark II Reverse Transcriptase (Invitrogen, America). For 5′ RACE, the RACE PCRs were performed by following the manufacturer’s instructions of 5′ RACE System for Rapid Amplification of cDNA Ends kit (Invitrogen, America). Resultant PCR products were cloned into pMD19-T Easy vector (Takara, Japan) and then sequenced (Majorbio, China). For 3′ RACE, the RACE PCRs were performed by following the manufacturer’s instructions of 3′RACE System for Rapid Amplification of cDNA Ends (Invitrogen, America). Resultant PCR products were cloned into pMD19-T Easy vector (Takara, Japan) and then sequenced (Majorbio, China).

Full-length sequences were spliced with DNAStar software. The specific primer sets (F and R, Table 1) were designed for identification of novel xylanolytic enzyme genes (*xyn-l1*, *xyn-l2*, *xyn-l4*, and *xys-l5*) with PCR amplification. The 25 µL PCR reaction contained 9.5 µL of ddH_2_O, 12.5 µL of premix Taq™ (Takara, Japan), 1 µL of primer-F (10 µM), 1 µL of primer-R (10 µM), and 1 µL of cDNA. PCR amplification was conducted on a thermal cycler (Eppendorf, Germany) under the following conditions: pre-heating at 95°C for 5 min, followed by 35 cycles of denaturation at 95°C for 1 min, annealing at 55°C for 1 min, and extension at 72°C for 1 min 30 s, and final extension at 72°C for 10 min. PCR products were electrophoresed on 1% agarose gel and stained with ethidium bromide dyestuff D1210 (Applygen, China). PCR amplicons of appropriate sizes were ligated into the pMD19-T Easy vector (Takara, Japan) for sequencing (Majorbio, China). Gene accession numbers were acquired using the NCBI Web BLAST service (http://www.ncbi.nlm.nih.gov/WebSub/?tool=genbank).

### Homology and Phylogeny Analyses of Novel Xylanolytic Enzymes

Homology between novel xylanolytic enzymes of *L. chartarum* SJTU59 and known xylanolytic enzymes in the NCBI GenBank database were analyzed using the NCBI Web BLAST service (protein blast; http://www.ncbi.nlm.nih.gov/BLAST/), GeneDoc software, and DNAStar software.

Phylogenetic tree was constructed based on the amino acid sequences of the four novel xylanolytic enzymes from *L. chartarum* SJTU59 and five representative industrial strains from the GenBank database using MEGA 4.0 software [25]. Amino acid alignments were done with DNAStar software.

## Results and Discussion

### Strain Identification with Morphology and Phylogeny

**Figure 1:**
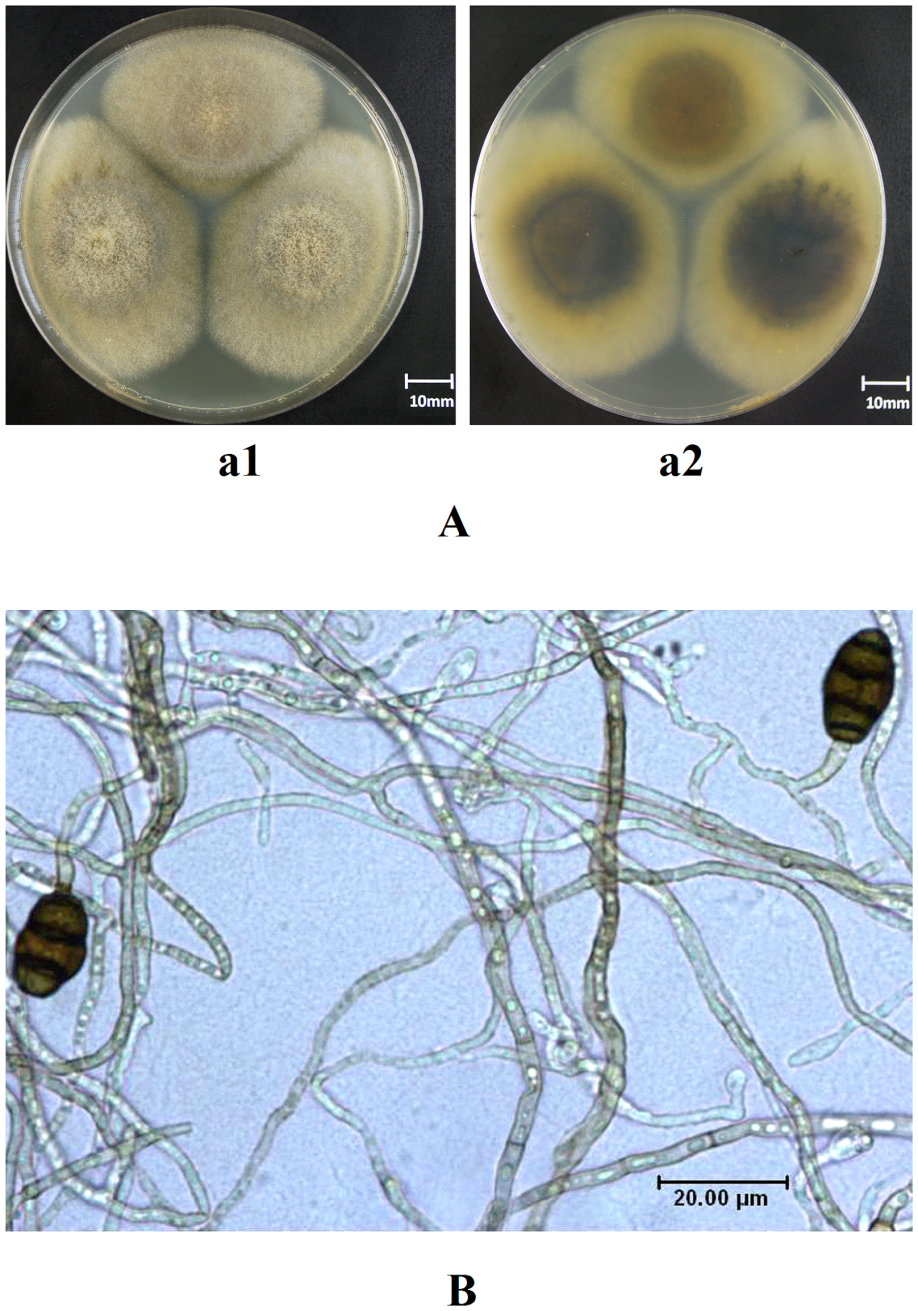
Morphological analysis of *L. chartarum* SJTU59. (**A**) Photograph of colonial morphology on PDA medium; a1, morphology of the front side; a2, morphology of the reverse side. (**B**) Micro-photograph of mycelia and spores.

Morphological observation showed that, cultured 7 d later, the front side of the velutinate colony of *L. chartarum* SJTU59 was gray brown, and the reversed side of it was tan. The colony with moderate thickness was 5.8 cm to 6.1 cm in diameter (Figure 1A). The transparent mycelium, with branch and septa, was smooth or slightly rough and the brown colored or colorless. Vegetative and spore hypha were 2.0 µm to 7.5 µm and 2.0 µm to 5.0 µm in diameter, respectively. Single spore mother cell, terminally or laterally born in spore hypha, was dentate or short cylindrical and measured (2.7 µm to 13.0 µm) × (2.0 µm to 3.0 µm). The dark brown conidium, which carried some parts of the spore mother cell at its base, was elliptic and obtuse on the apex. Three transverse septum and 0 to 1 longitudinal septa were usually observed in the conidium, and measured (10.0 µm to 19.0 µm) × (8.0 µm to 12.5 µm) (Figure 1B).

Electrophoretic gel images showed that the conserved ITS (652 bp, KC879283) and EF-1α (373 bp) sequences of *L. chartarum* SJTU59 were successfully amplified using specific primer sets (Figure S1). Nucleotide alignment showed that nrDNA ITS of *L. chartarum* SJTU59 shared 100% similarity with that of *L. chartarum* CY233 (HQ608046) (Figure S2A). The EF-1α gene of *L. chartarum* SJTU59 had 100% similarity to that of *L. chartarum* L119 (Figure S2B).

**Figure 2:**
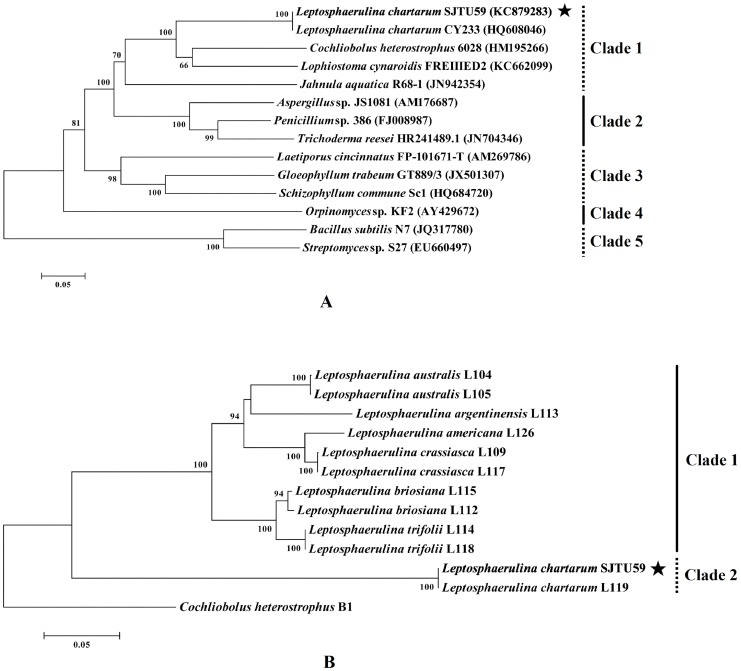
Phylogeny analysis of *L. chartarum* SJTU59. (**A**) Phylogenetic tree based on nrDNA ITS sequence of *L. chartarum* SJTU59 (bold and marked with an asterisk) and some related sequences of representative strains from the GenBank database. (**B**) Phylogenetic tree based on EF-1α sequences of *L. chartarum* SJTU59 (bold and marked with an asterisk) and its closely related species and outgroup retrieved from the literature.

Phylogenetic analysis based on nrDNA ITS (incl. two 16S rDNA sequences) consisted of five distinct clades with high bootstrap support. As bacteria, *Streptomyces* sp. S27 and *Bacillus subtilis* N7 in clade 5 were grouped out from fungi (kingdom) in clade 1 to clade 4 with 100% bootstrap support. With the strong bootstrap supports (81% and 98%), fungi belonging to the Ascomycota (phylum) in clade 1 and clade 2, were separated from that of other phyla in clade 3 and clade 4. And with 100% bootstrap support, fungi belonging to the Dothideomycetes (class) in clade 1 were grouped out from that of different classes in clade 2. Within clade 1, *Jahnula aquatica* R68-1 and *L. chartarum* SJTU59 belonged to the same class (Dothideomycetes) but to different order. Identical with *L. chartarum* CY233 (100% bootstrap support), *L. chartarum* SJTU59 shared the same family (Didymellaceae) but different genus with *Cochliobolus heterostrophus* 6028, and was in the same order (Pleosporales) but different family with *Lophiostoma cynaroidis* FREIIIED2 (Figure 2A). In conclusion, the novel strain could be identified as a fungus belonging to Ascomycota, Dothideomycetes, Pleosporales, Didymellaceae, Leptosphaerulina and Chartarum, and was named as SJTU59.

Furthermore, phylogenetic analysis of the EF-1α sequence showed that *L. chartarum* SJTU59 was identical with *L. chartarum* L119 (clade 2). However, the closely related species in clade 1 were grouped out from *L. chartarum* SJTU59 and L119 with 100% bootstrap support, which suggested that *L. chartarum* was distinct from the closely related species. *Cochliobolus heterostrophus* B1 (anamorph: *Bipolaris maydis*) shared the same family (Pleosporaceae) with *Leptosphaerulina* (Figure 2B).

### Xylanolytic Enzyme Activities with Different Carbon Sources

**Figure 3:**
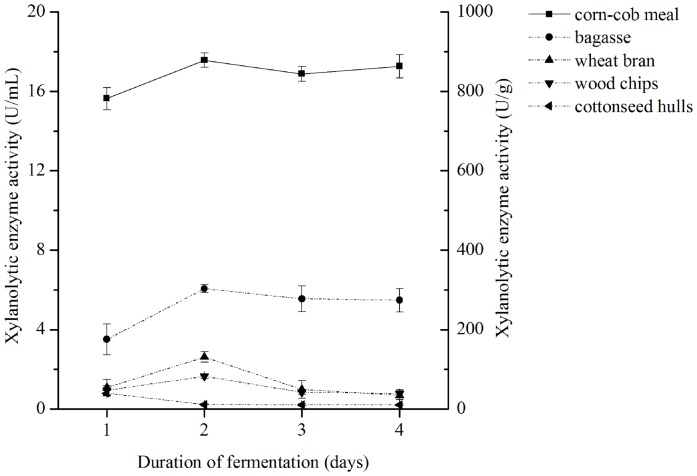
Xylanolytic enzyme activity produced by *L. chartarum* SJTU59. Data were expressed as mean values of triplicate measurements with two kinds of units (U/mL or U/g substrate). Bars indicated standard deviations.

Data showed that xylanolytic enzyme activity was significantly higher in the fermentation broth of *L. chartarum* SJTU59 with corncob meal as the carbon source compared to those with other agro-industrial wastes. The highest xylanolytic enzyme activities were 17.566±0.358 U/mL (equivalent to 878.307±17.913 U/g substrate) with corncob meal, 6.066±0.203 U/mL (equivalent to 303.317±10.136 U/g substrate) with sugarcane bagasse, 2.614±0.286 U/mL (equivalent to 130.717±13.409 U/g substrate) with wheat bran, 1.655±0.124 U/mL (equivalent to 82.773±6.193 U/g substrate) with wood chips, and 0.799±0.144 U/mL (equivalent to 39.965±7.192 U/g substrate) with cottonseed hulls (Figure 3).

Although fungal strains are commonly reported as major microbial producers of xylanolytic enzymes, including members of *Trichoderma*, *Aspergillum*, and *Penicillium*, this study is the first demonstration of the high-level production of stable xylanolytic enzymes by a novel strain, *L. chartarum* SJTU59 [1]. In addition, our results showed that the highest production of xylanolytic enzymes from *L. chartarum* SJTU59 under un-optimized conditions was comparable to values reported in known xylanolytic enzyme-producing *T. longibrachiatum*, *A. fumigatus*, *P. janthinellum*, and *B. subtilis* under optimized conditions [15], [19]–[21]. This finding expands the range of microbial resource for screening of high-yield xylanolytic enzyme producers, thus contributing to industrial and agricultural production of the enzyme preparation.

The following experiments were carried out using the corncob-containing fermentation broth because it demonstrated high efficiency in xylanolytic enzyme production.

### Xylanolytic Enzyme Activities under Different PH, Temperature, or chemical conditions

**Figure 4:**
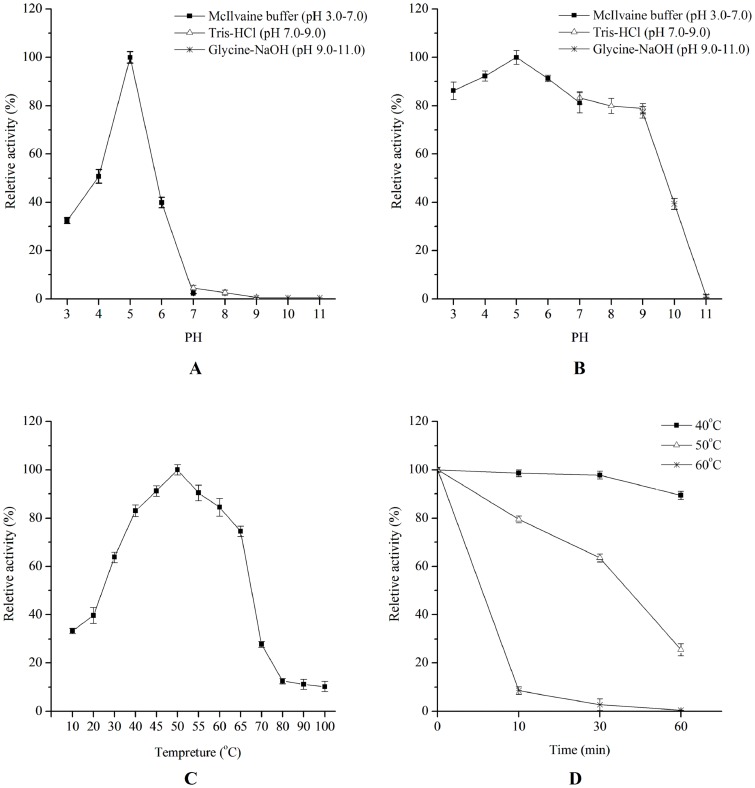
Effects of pH and temperature on the activity and stability of xylanolytic enzymes from *L. chartarum* SJTU59. (**A**) pH effect on xylanolytic enzyme activity determined in the McIlvaine buffer (pH 3.0 to pH 7.0), Tris-HCl (pH 7.0 to pH 9.0), and Glycine-NaOH solution (pH 9.0 to pH 11.0) at 50°C. (**B**) pH stability of xylanolytic enzyme determined at 50°C after 12 h of pre-incubation in the McIlvaine buffer (pH 3.0 to pH 7.0), Tris-HCl (pH 7.0 to pH 9.0) or Glycine-NaOH solution (pH 9.0 to pH 11.0) at 4°C. (**C**) Temperature effect on xylanolytic enzyme activity determined in sodium acetate buffer (0.1 M, pH 5.0, 10°C to 100°C). (**D**) Thermostability of xylanolytic enzymes determined at 50°C after pre-incubation in sodium acetate buffer (pH 5.0) at 40, 50, or 60°C without substrate.

Over the pH range of 3.0 to 11.0, xylanolytic enzymes produced by *L. chartarum* SJTU59 had the highest activity at pH 5.0 and retained more than 40% of peak enzyme activity between pH 4.0 and pH 6.0 (Figure 4A). After pre-incubation at pH 3.0 to pH 9.0 for 12 h, the xylanolytic enzymes retained more than 80% of the initial activity, whereas those pre-incubated at pH 10.0 to pH 11.0 showed substantial decrease in xylanolytic enzyme activity (Figure 4B). Hence, enzyme activity preferentially occur under slightly acidic conditions (pH 4.0 to pH 6.0), but is still maintained under slightly alkaline conditions (pH 7.0 to pH 9.0).

Over the temperature range of 10°C to 100°C, xylanolytic enzymes produced by *L. chartarum* SJTU59 had the highest activity at 50°C and retained 33.3%, 63.7%, and 74.5% of the peak enzyme activity at 10°C, 30°C, and 65°C, respectively (Figure 4C). Enzyme activity rapidly declined at temperatures exceeding 70°C and only retained 10.2% of peak xylanolytic enzyme activity at 100°C. Evaluation of enzyme thermostability showed that pre-incubation of the xylanolytic enzymes at 40°C for 10 min to 60 min resulted in less than 10% loss of the initial enzyme activity, whereas pre-incubation at 60°C for 10 min to 60 min led to over 90% loss of enzyme activity. After pre-incubation at the 50°C (optimal temperature) for 30 or 60 min, xylanolytic enzymes retained over 60% and nearly 30% of initial activity, respectively (Figure 4D). These results indicate that xylanolytic enzymes produced by *L. chartarum* SJTU59 have relatively high thermostability.

In the presence of specific metal ions or chemical compounds, the activity of xylanolytic enzymes showed varied trends (Table 2). Enzyme activity was strongly enhanced by 10 mM of Mg^2+^, 1 mM or 10 mM of Co^2+^, or 10 mM of Al^3+^ (over 10% increase in relative activity), and significantly inhibited by EDTA or SDS at 1 or 10 mM (over 90% decrease in relative activity). Cu^2+^ greatly inhibited enzyme activity only at high concentration (10 mM). Other metal irons (at 1 mM or 10 mM) had minor positive or negative effects on the enzyme activity.

**Table 2:** Effects of metal ions and chemical compounds on xylanolytic enzyme activity.

Metal ions and chemical compounds	Relative activity of xylanolytic enzyme (%)^a^	
	1 mM	10 mM
Control	100.000	100.000
Li^+^	97.813	105.243
Na^+^	97.998	101.221
K^+^	104.097	101.676
Ca^2+^	105.752	100.485
Mg^2+^	101.174	130.262
Cu^2+^	98.324	45.751
Zn^2+^	98.696	91.729
Ni^2+^	96.774	92.818
Fe^3+^	99.187	94.227
Cr^3+^	109.497	108.752
Co^2+^	124.776	154.135
Al^3+^	100.010	111.729
Ba^2+^	100.004	100.903
EDTA	8.241	2.779
SDS	5.692	1.566

a The data represent the mean to untreated control samples from triplicate measurements (SD ≤5%).

Therefore, associated enzyme preparations from *L. chartarum* SJTU59 can be modified and enhanced for use in industrial applications in pulp/paper leaching at high temperatures (55°C to 70°C) and alkaline pH (pH 7.0 to pH 9.0), or acid pre-treatment in bioconversion processes or detergent application at high temperatures [1]. The xylanolytic enzyme solution with high resistance to a variety of metal ions can be used in deinking and metal-polluted sewage treatment [32]. Given the initially high production and stable properties of xylanolytic enzymes under various adverse conditions, further improvement of xylanolytic enzyme production by *L. chartarum* SJTU59 can be done via optimization of fermentation conditions [14], [19]. Alternatively, site-directed and random mutagenesis can be used to modify the enzymes using protein engineering techniques [33].

### Hydrolytic Product Compositions of Xylanolytic Enzymes

**Figure 5:**
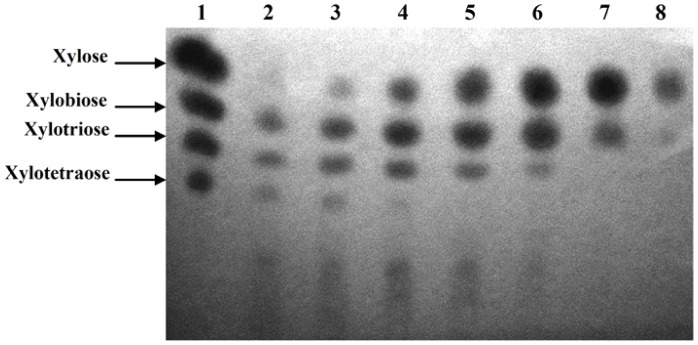
TLC analysis of the hydrolytic products of xylan and xylooligosaccharides by xylanolytic enzymes of *L. chartarum* SJTU59. Lane 1, marker of xylose and xylooligosaccharides; Lanes 2 to 8, crude enzyme solution +1% beechwood xylan substrate in 0.1M sodium acetate buffer (pH 5.0) at 50°C for 24 h (Lane 2, 20 µL +980 µL; Lane 3, 30 µL +970 µL; Lane 4, 50 µL +950 µL; Lane 5, 200 µL +800 µL; Lane 6, 300 µL +700 µL; Lane 7, 500 µL +500 µL; Lane 8, 800 µL +200 µL). The composition of hydrolytic products gradually changed with the proportions of enzymes and substrate.

TLC analysis showed that in the presence of sufficient substrate, hydrolytic products of beechwood xylan degraded by xylanolytic enzymes mainly contained xylooligosaccharides (Figure 5), an emerging prebiotic in applications of food industry [7]. Thus, *L. chartarum* SJTU59 has great potential for large-scale production of xylooligosaccharides by optimization of the reaction system and fermentation conditions, including the reaction time, temperature, and/or pH. With an increase in volume of enzyme solution and a decrease in volume of xylan substrate, the degree of xylan hydrolysis gradually increased, and the main hydrolytic products changed to xylose with a small amount of xylobiose (Figure 5).

In summary, the results indicate that the xylanolytic enzyme system of *L. chartarum* SJTU59 contains at least two types of xylanolytic enzymes, i.e., endo-xylanase that cleaves internal xylosidic linkages on the backbone of xylan, and xylosidase that cleaves glycoside linkage of xylooligosaccharides (or exo-xylanase that cleaves external xylosidic linkages on the backbone of xylan).

### Assays of Tissue Necrosis and Oxidative Burst

**Figure 6:**
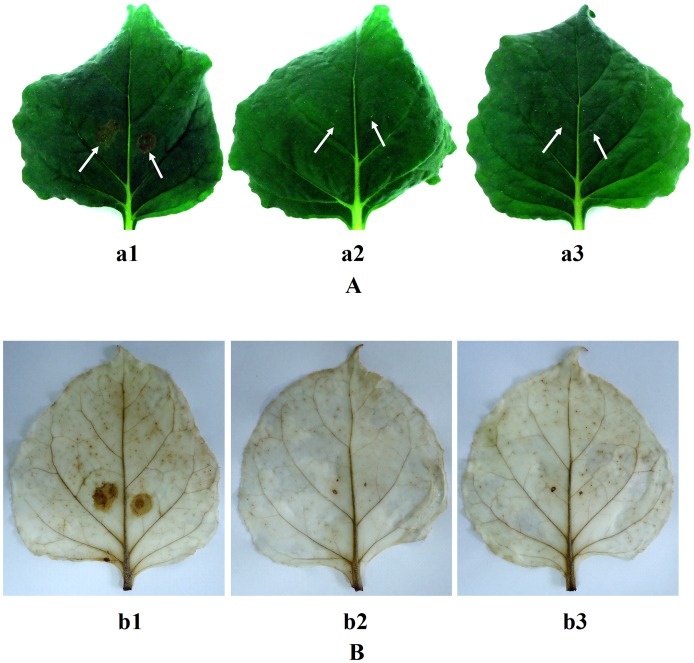
Tissue necrosis in tobacco plant and DAB staining analyses. (**A**) Photographs of tissue necrosis in tobacco leaves after inoculation for 24 h; a1, tobacco leaf injected with xylanolytic enzyme preparation of *L. chartarum* SJTU59; a2, tobacco leaf injected with supernatant of blank broth (control); a3, tobacco leaf injected with distilled water (control). (**B**) Photographs of DAB staining in tobacco leaves after inoculation for 24 h; b1, tobacco leaf injected with xylanolytic enzyme preparation of *L. chartarum* SJTU59; b2, tobacco leaf injected with supernatant of blank broth (control); b3, tobacco leaf injected with distilled water (control).

At 12 h after inoculation, necrotic lesions were seen on tobacco leaves injected with xylanolytic enzyme preparation from *L. chartarum* SJTU59, but no necrotic lesions were seen on the leaves treated with blank broth and distilled water. This contrasting result was most apparent at 24 h (Figure 6 A). In addition, inoculated sites and certain distant sites of tobacco leaves injected with xylanolytic enzyme preparation from *L. chartarum* SJTU59 could be stained strongly by DAB, whereas those in tobacco leaves injected with blank broth and distilled water showed nearly no staining (Figure 6 B).

As DAB staining reflects H_2_O_2_ accumulation, results illustrate that oxidative burst in tobacco leaves is induced by xylanolytic enzyme preparation from *L. chartarum* SJTU59 [29]. H_2_O_2_ and O_2_^-^ from oxidative burst are correlated with hypersensitive response (HR) and cause general cell death [34], [35]. In addition, as a typical second messenger, H_2_O_2_ directly and indirectly (such as via HR) regulates the innate immune system and systemic acquired resistance (SAR) of plants, to enhance plant defense against adversity and disease [36], [37].

For industrial and agricultural uses, further study at the molecular level on the xylanolytic enzyme genes of *L. chartarum* SJTU59 is important.

### Sequence Analysis of Novel Xylanolytic Enzyme Genes

PCR assay with GH 10-targeting degenerate primers yielded four bands on the electrophoretic gel image (Figure S3A). DNA sequencing (data not shown) and sequence alignment on NCBI website showed that the two bands may represent the conserved regions (322 bp and 262 bp) of two endo-β-1,4-xylanase genes in the GH family 10, designated *xyn-l1* (intron: 57 bp) and *xyn-l2* (non-intron), respectively. PCR assay with GH 11-targeting degenerate primers yielded one band on the electrophoretic gel image (Figure S3B). However, DNA sequencing and sequence alignment on NCBI website showed that the PCR amplicons may contain conserved regions (273 bp and 266 bp) of two endo-β-1,4-xylanase genes in the GH family 11, designated *xyn-l3* (intron: 60 bp) and *xyn-l4* (intron: 53 bp), respectively. PCR assay with GH43-targeting degenerate primers yielded one band on the electrophoretic gel image (Figure S3C). DNA sequencing (data not shown) and sequence alignment on NCBI website showed that the band may represent the conserved region (613 bp) in a β-xylosidase gene in the GH family 43, designated *xys-l5* (non-intron).

PCR identification of four novel full-length genes of xylanolytic enzymes, including *xyn-l1*, *xyn-l2*, *xyn-l4*, and *xys-l5* acquired via RACE, all yielded one specific band with the appropriate size on electrophoretic gel images (Figure S4). DNA sequences (data not shown) and sequence alignments with RACE results showed that the sequences of *xyn-l1* (KF367459, 1,062 bp), *xyn-l2* (KF305938, 1,152 bp), *xyn-l4* (KF305939, 693 bp), and *xys-l5* (KF305940, 1,020 bp) were all correct. However, *xyn-l3* was inferred to be a pseudogene or low-expression abundance gene, as at RNA level its conserved, 5′ RACE and 3′ RACE sequences all could not be obtained.

Results prove that *L. chartarum* SJTU59 has a complex xylanolytic enzyme system, thus ensuring high-efficiency hydrolysis of xylan. Considering the potential limitation of selected primers [30], [31], we believe that undetected gene(s) are possibly present in the xylanolytic enzyme system of *L. chartarum* SJTU59. To identify other novel xylanolytic enzyme genes, future studies will be conducted on primer design in consideration of other less-known GH families, e.g., GH 5, 7, and 8.

### Homology and Function Analyses of Novel Xylanolytic Enzymes

**Figure 7:**
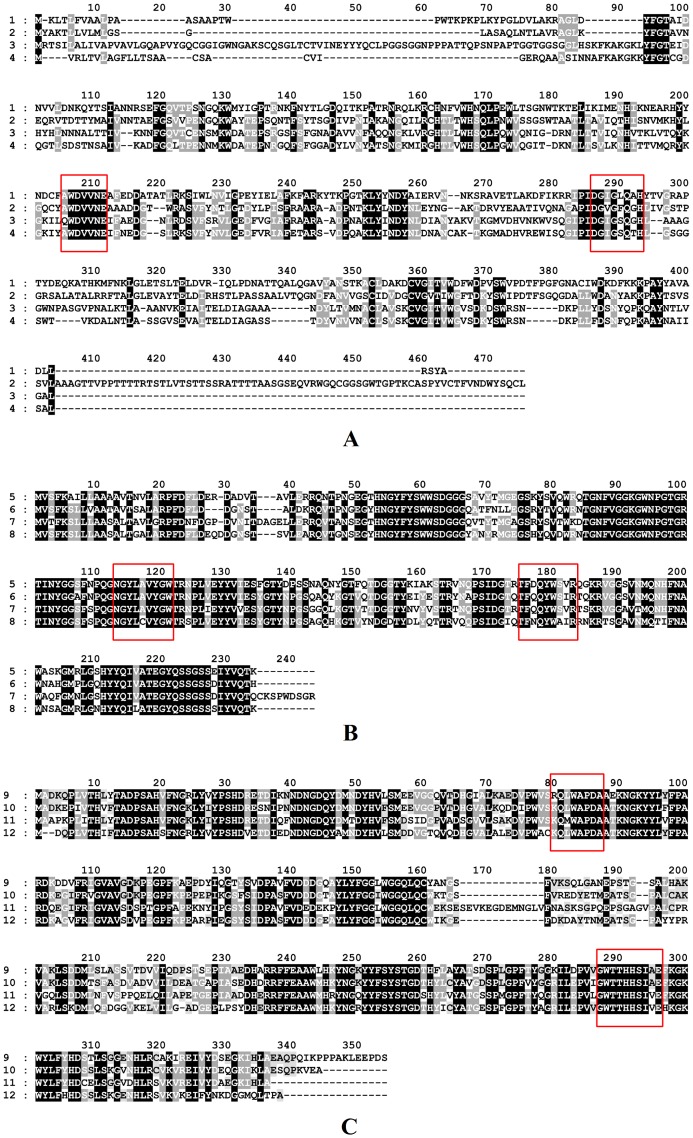
Amino acid sequence analysis of the novel xylanolytic enzymes in *L. chartarum* SJTU59. (**A**) 1 (XYN-L1) and 3 (XYN-L2) are the amino acid sequences encoded by the two novel xylanase genes (*xyn-l1* and *xyn-l2*); and 2 and 4 are the homologous sequences of XYN-L1 and XYN-L2 in the GenBank database, respectively. (**B**) 5 (XYN-L4) is the amino acid sequence coded by the novel xylanase gene (*xyn-l4*); and 6, 7, and 8 are the homologous sequences of XYN-L4 in the GenBank database. (**C**) 9 (XYS-L5) is the amino acid sequence encoded by the novel xylosidase gene (*xys-l5*); and 10, 11, and 12 are the homologous sequences of XYS-L5 in the GenBank database. Identical and similar amino acids are highlighted in solid-black and gray colors, respectively, and red boxes represent the conservative regions.

Amino acid homology analyses showed that XYN-L1 shared 41.7% homology with that of the endo-β-1,4-xylanase (ELA24762, GH family 10) from *Colletotrichum gloeosporioides* Nara gc5, and XYN-L2 shared 63.8% homology with that of the endo-β-1,4-xylanase (AFD63136, GH family 10) from *Aspergillus terreus* (Figure 7A).

XYN-L4 shared 78.9% homology with that of the endo-β-1,4-xylanase (ADW78258, GH family 11) from *Chaetomium* sp. CQ31, 76.5% homology with that of the endo-β-1,4-xylanase (XP_003006739, GH family 11) from *Verticillium albo-atrum* VaMs.102 and 73.9% homology with that of the endo-β-1,4-xylanase (EMT73821, GH family 11) from *Fusarium oxysporum* f. sp. *cubense* race 4 (Figure 7B).

XYS-L5 shared 75.7% homology with that of the β-xylosidase (XP_001932957, GH family 43) from *Pyrenophora tritici-repentis* Pt-1C-BFP, 68.1% homology with that of the β-xylosidase (EKD19100, GH family 43) from *Marssonina brunnea* f. sp. *multigermtubi* MB_m1, and 69.2% homology with that of the β-D-xylanase (AFX58964, GH family 43) from *Talaromyces purpurogenus* (Figure 7C).

In accordance with the CAZy classification system, members of the same GH family share similar structural and functional roles [3]. Previously, the majority of (hyper) thermophilic, psychrophilic, alkaliphilic, and/or acidophilic xylanases were identified in GH families 10 and 11 [1]. A GH family 10-related xylanase, Xylk, is activated by the presence of a certain amount of Ca^2+^, Co^2+^, and Fe^2+^ [38]. We propose that the xylanase stability and resistance of *L. chartarum* SJTU59 in various temperatures, pH, or chemical conditions (Figure 4 and Table 2) are related to the existence of the three endo-β-1,4-xylanases of GH families 10 and 11. In addition, xylanase II from GH family 11 induces necrotic lesions and ethylene biosynthesis in tobacco, thereby potentially improving the resistance and immunity of plants to diseases [39]. We infer that necrotic lesions and oxidative burst (Figure 6) of tobacco may be related to endo-β-1,4-xylanase of GH families 11 from *L. chartarum* SJTU59. However, little is known regarding the structural or functional roles of GH family 43 members. We are uncertain about the properties of β-xylosidase detected in *L. chartarum* SJTU59.

### Phylogeny Analysis of Novel Xylanolytic Enzymes

**Figure 8:**
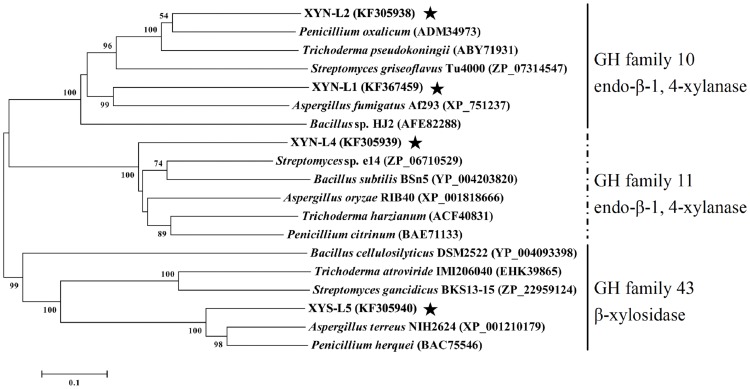
Phylogeny analysis of the novel xylanolytic enzymes in *L. chartarum* SJTU59. Phylogenetic tree based on the amino acid sequences of four novel xylanolytic enzymes (bold and marked with an asterisk) detected in *L. chartarum* SJTU59 and those of the homologs as well as five well-known microbial producers of xylanases retrieved from the GenBank database.

The amino acid sequence homologies among novel xylanolytic enzymes detected in *L. chartarum* SJTU59 and known xylanolytic enzymes in five representative industrial strains of xylanolytic enzyme producer were shown in Table 3 to Table 5. Overall, the homologies of deduced amino acid sequences were 27.4% to 61.4% between XYN-L1 to XYN-L2 and known endo-β-1,4-xylanases from the GH family 10, 47.3% to 63.1% between XYN-L4 and known endo-β-1,4-xylanases from the GH family 11, and 14.2% to 70.3% between XYS-L5 and known β-xylosidases from the GH family 43 (Figure 8). Hence, the amino acid sequence homologies and phylogenetic tree results indicate that *L. chartarum* SJTU59 contains at least four novel genes that encode three novel xylanases and one novel xylosidase, including two endo-β-1,4-xylanases of the GH family 10, one endo-β-1,4-xylanase of the GH family 11, and one β-xylosidase of the GH family 43.

**Table 3:** Amino acid sequence homologies among novel xylanases in GH family 10 from *L. chartarum* SJTU59 and known xylanases from five representative industrial strains.

Number allocated to strain (GH 10, endo-β-1,4-xylanase)								
1	2	3	4	5	6	7	Number	Strain
–	32.7	32.4	42.1	35.5	32.9	27.4	1	XYN-L1 (KF367459)
	–	55.1	43.5	61.4	43.6	31.7	2	XYN-L2 (KF305938)
		−	39.2	60.6	41.4	30.0	3	*Trichoderma pseudokoningii* (ABY71931)
			−	44.9	34.3	32.9	4	*Aspergillus fumigatus* Af293 (XP_751237)
				−	44.5	33.4	5	*Penicillium oxalicum* (ADM34973)
					−	35.3	6	*Streptomyces griseoflavus* Tu4000 (ZP_07314547)
						−	7	*Bacillus* sp. HJ2 (AFE82288)

**Table 4:** Amino acid sequence homologies among novel xylanase in GH family 11 from *L. chartarum* SJTU59 and known xylanases from five representative industrial strains.

Number allocated to strain (GH 11, endo-β-1,4-xylanase)							
1	2	3	4	5	6	Number	Strain
−	54.5	54.8	53.9	55.6	51.5	1	XYN-L4 (KF305939)
	−	55.9	63.1	54.9	47.3	2	*Trichoderma harzianum*(ACF40831)
		−	59.0	63.1	51.5	3	*Aspergillus oryzae* RIB40(XP_001818666)
			−	61.1	50.5	4	*Penicillium citrinum*(BAE71133)
				−	61.8	5	*Streptomyces* sp. e14(ZP_06710529)
					−	6	*Bacillus subtilis* BSn5(YP_004203820)

**Table 5:** Amino acid sequence homologies among novel xylosidase in GH family 43 from *L. chartarum* SJTU59 and known xylosidases from five representative industrial strains.

Number allocated to strain (GH 43, β-xylosidase)							
1	2	3	4	5	6	Number	Strain
−	25.4	70.3	68.4	23.0	21.3	1	XYS-L5 (KF305940)
	−	22.5	21.4	59.6	14.2	2	*Trichoderma atroviride* IMI206040 (EHK39865)
		−	75.5	20.4	22.3	3	*Aspergillus terreus* NIH2624 (XP_001210179)
			−	20.2	19.6	4	*Penicillium herquei* (BAC75546)
				−	14.2	5	*Streptomyces gancidicus* BSK13-15 (ZP_22959124)
					−	6	*Bacillus cellulosilyticus* DSM2522 (YP_004093398)

In summary, for industrial and agricultural uses, cloning, expression, and characterization of high-yield xylanolytic enzymes from *L. chartarum* SJTU59 are critical to improve current understanding of their enzyme activities and functions [28]. Cloned and expressed xylanolytic enzymes can be compared with known xylanolytic enzymes from recognized microbial producers to identify the advantage of novel xylanolytic enzymes detected in *L. chartarum* SJTU59. Based on the present study, novel xylanolytic enzyme-producing engineered strain(s) can be developed via transgenic approach or overexpression in yeast [40].

## Conclusion

This study was the first demonstration of high-level xylanolytic enzyme production by a novel fungus, designated *L. chartarum* SJTU59. The enzyme solution was relatively stable over a wide range of pH (pH 3.0 to pH 9.0) and temperature (40°C to 65°C) while showing high resistance to the majority of metal ions tested. These characters of xylanolytic enzymes are suitable for pulp/paper leaching, deinking and metal-polluted sewage treatment in industrial applications. Analysis of the hydrolytic products of xylan showed that in the presence of sufficient xylan substrate, xylooligosaccharides, an emerging prebiotic in applications of food industry, were mainly produced. Excluding industrial applications, the xylanolytic enzyme fermentation supernatant of *L. chartarum* SJTU59 may be related to enhanced plant defense against adversity and disease. *L. chartarum* SJTU59 possessed a complex xylanolytic enzyme system: two endo-β-1,4-xylanases of the GH family 10, one endo-β-1,4-xylanase of the GH family 11, and one β-xylosidase of the GH family 43, respectively. Future studies will be conducted to characterize the properties of individual xylanolytic enzymes from *L. chartarum* SJTU59 via expression them in *Escherichia coli*, respectively, and construct genetically engineered strains with the xylanase-encoding gene(s) for potential use in industrial and agricultural applications.

## Supporting Information

Figure S1 **Electrophoretic gel images of PCR amplification of ITS and EF-1α sequences from** ***L. chartarum*** **SJTU59.** **(A)** ITS amplification; M, Marker DL2000 (Takara, Japan); 1, band of ITS. **(B)** EF-1α amplification; M, Marker DL2000 (Takara, Japan); 1, band of EF-1α.
(TIF)

Figure S2 **Molecular identification of** ***L. chartarum*** **SJTU59 with nrDNA ITS and EF-1α sequences.** **(A)** Alignment of nrDNA ITS sequences from *L. chartarum* SJTU59 and CY233; 1, nrDNA ITS sequence of *L. chartarum* SJTU59; 2, nrDNA ITS sequence of *L. chartarum* CY233. **(B)** Alignment of EF-1α sequences from *L. chartarum* SJTU59 and L119; 1, EF-1α sequence of *L. chartarum* SJTU59; 2, EF-1α sequence of *L. chartarum* L119.
(TIF)

Figure S3 **Electrophoretic gel images of PCR amplification of conserved regions from five novel xylanolytic enzyme genes.** **(A)** PCR amplification of the conserved regions of two novel xylanase genes (*xyn-l1* and *xyn-l2*) belonging to the GH family 10; M, DL2000 (Takara, Japan); GH 10, bands of amplification; 1 and 2 are the bands of *xyn-l1* and *xyn-l2*. **(B)** PCR amplification of the conserved regions of two novel xylanase genes (*xyn-l3* and *xyn-l4*) of the GH family 11; M, DL2000 (Takara, Japan); GH11, bands of amplification; 3 and 4 are the bands of *xyn-l3* and *xyn-l4*. **(C)** PCR amplification of the conserved region of one novel xylosidase genes (*xys-l5*) of the GH family 43; M, DL2000 (Takara, Japan); GH43, the band of amplification; 5 is the band of *xys-l5*.
(TIF)

Figure S4 **Electrophoretic gel images of PCR amplification of the CDS belonging to four novel xylanolytic enzyme genes.** **(A)** PCR amplification of *xyn-l1*; M, 100 bp DNA Ladder (Real-Times, China); 1, the band of *xyn-l1*. **(B)** PCR amplification of *xyn-l2*; M, 100 bp DNA Ladder (Real-Times, China); 1, the band of *xyn-l2*. **(C)** PCR amplification of *xyn-l4*; M, 100 bp DNA Ladder (Real-Times, China); 1, the band of *xyn-l4*. **(D)** PCR amplification of *xys-l5*; M, 100 bp DNA Ladder (Real-Times, China); 1, the band of *xys-l5*.
(TIF)
