# Supplementary figures and images for: Identification of a Novel Fungus, Leptosphaerulina chartarum SJTU59 and Characterization of Its Xylanolytic Enzymes
Source: PLoS One. 2013 Sep 9;8(9):e73729. doi: 10.1371/journal.pone.0073729 (PMC3767624; doi:10.1371/journal.pone.0073729)

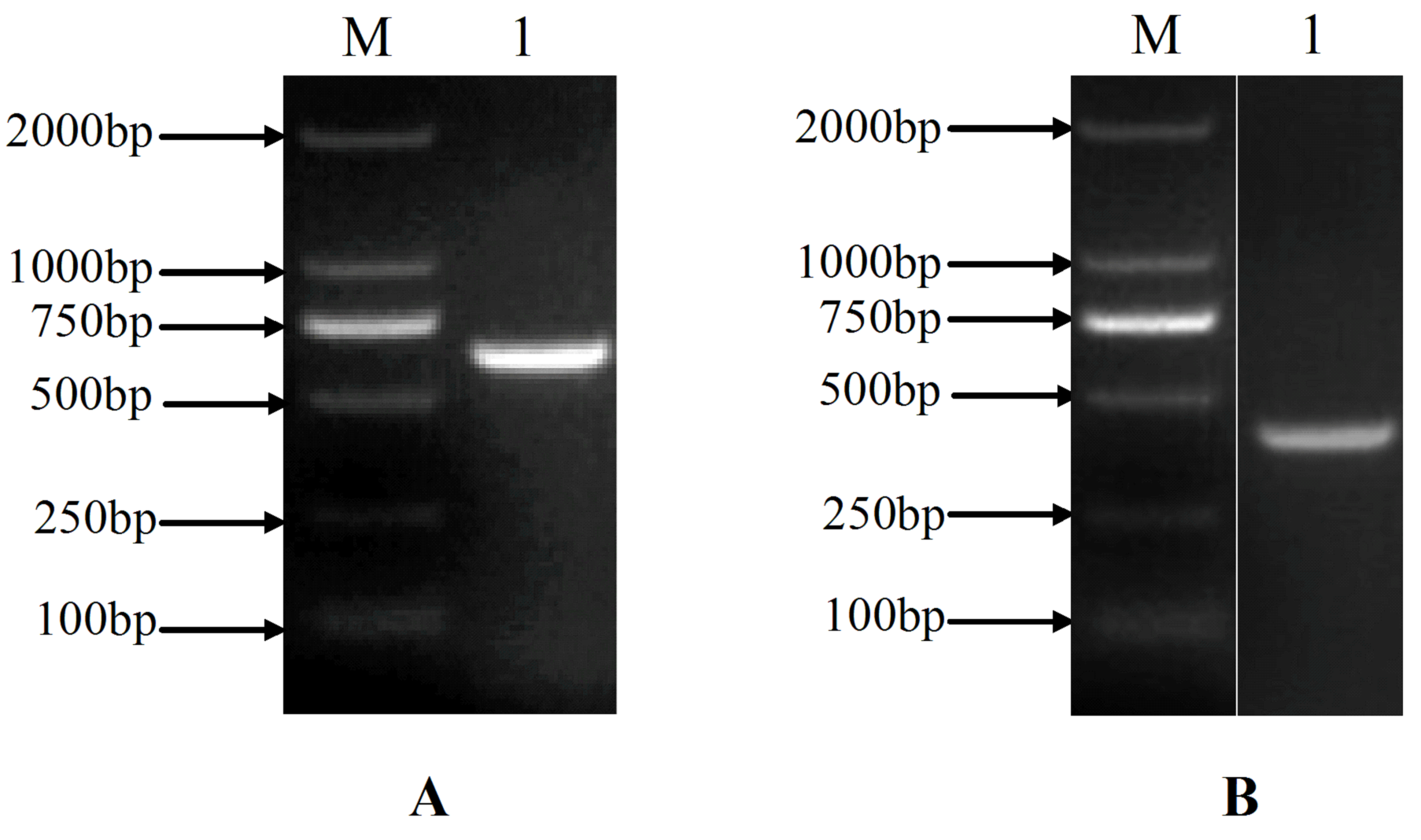

Supplement: Figure S1 — Electrophoretic gel images of PCR amplification of ITS and EF-1α sequences from L. chartarum SJTU59. (A) ITS amplification; M, Marker DL2000 (Takara, Japan); 1, band of ITS. (B) EF-1α amplification; M, Marker DL2000 (Takara, Japan); 1, band of EF-1α. (TIF) [file pone.0073729.s001.tif]

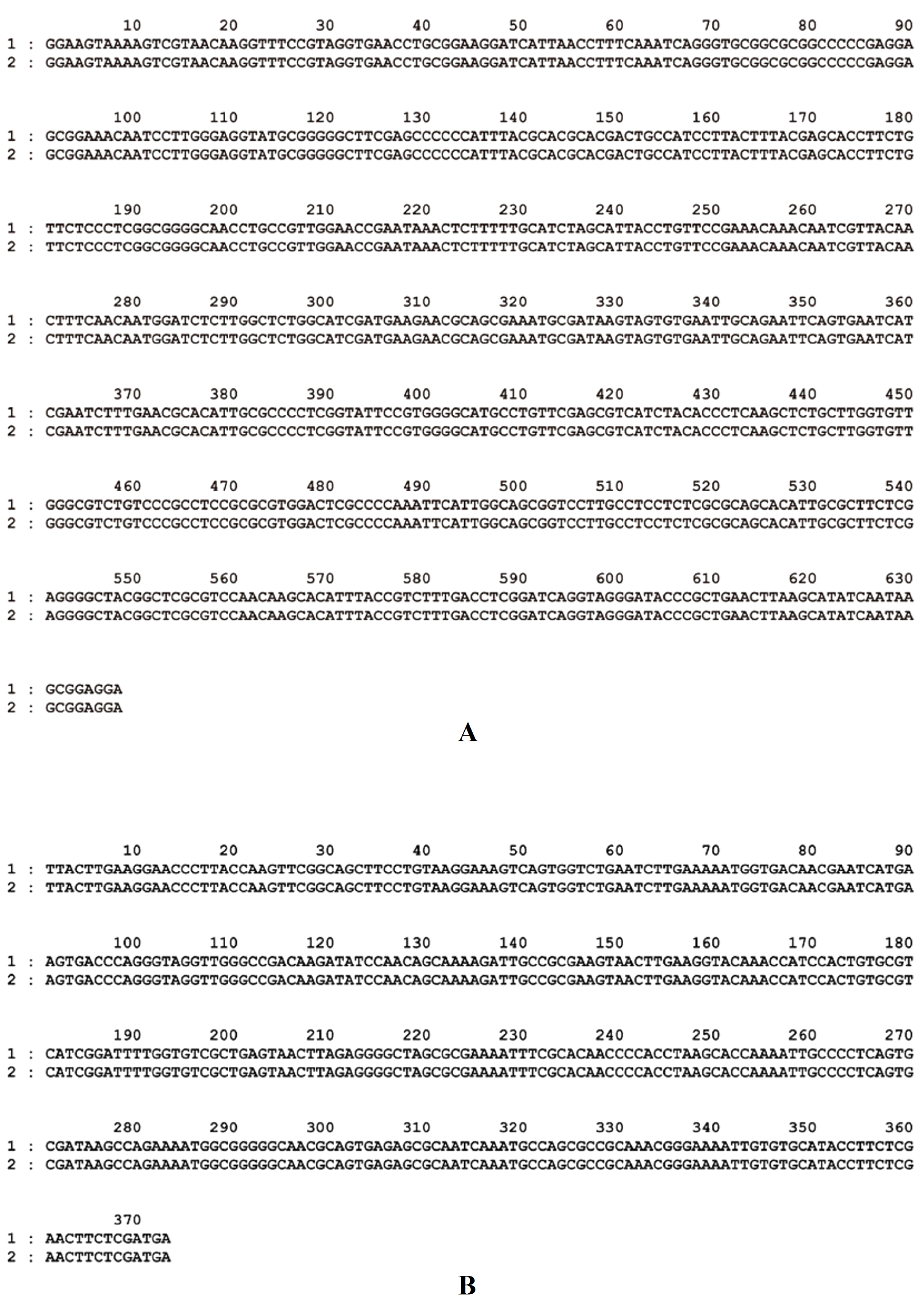

Supplement: Figure S2 — Molecular identification of L. chartarum SJTU59 with nrDNA ITS and EF-1α sequences. (A) Alignment of nrDNA ITS sequences from L. chartarum SJTU59 and CY233; 1, nrDNA ITS sequence of L. chartarum SJTU59; 2, nrDNA ITS sequence of L. chartarum CY233. (B) Alignment of EF-1α sequences from L. chartarum SJTU59 and L119; 1, EF-1α sequence of L. chartarum SJTU59; 2, EF-1α sequence of L. chartarum L119. (TIF) [file pone.0073729.s002.tif]

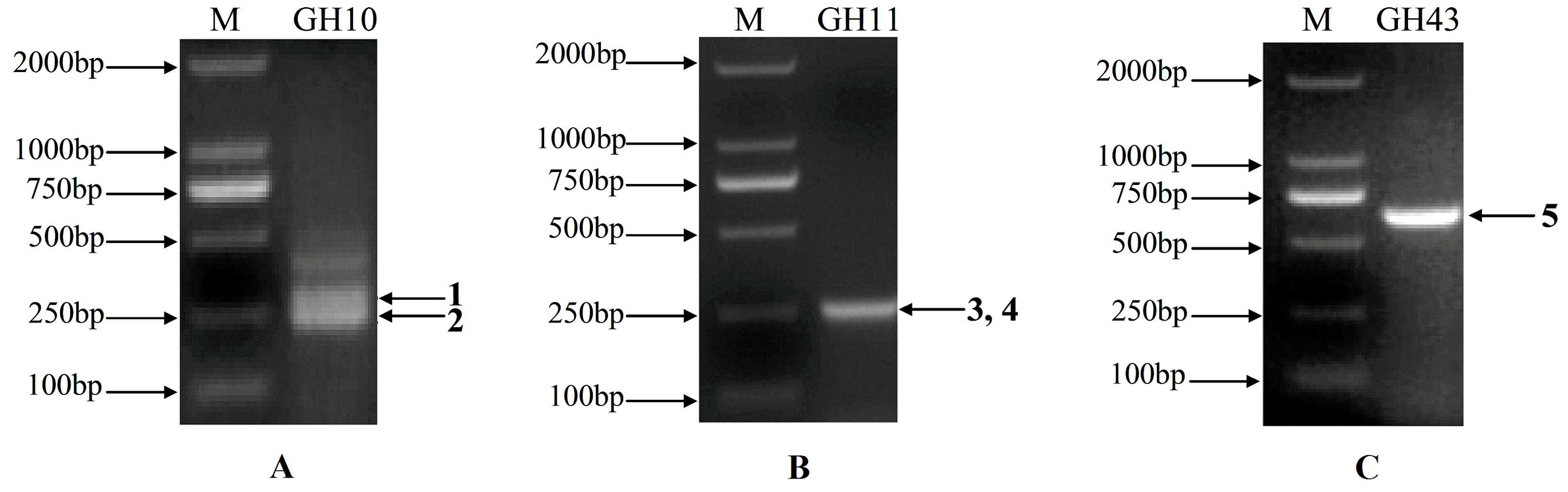

Supplement: Figure S3 — Electrophoretic gel images of PCR amplification of conserved regions from five novel xylanolytic enzyme genes. (A) PCR amplification of the conserved regions of two novel xylanase genes (xyn-l1 and xyn-l2) belonging to the GH family 10; M, DL2000 (Takara, Japan); GH 10, bands of amplification; 1 and 2 are the bands of xyn-l1 and xyn-l2. (B) PCR amplification of the conserved regions of two novel xylanase genes (xyn-l3 and xyn-l4) of the GH family 11; M, DL2000 (Takara, Japan); GH11, bands of amplification; 3 and 4 are the bands of xyn-l3 and xyn-l4. (C) PCR amplification of the conserved region of one novel xylosidase genes (xys-l5) of the GH family 43; M, DL2000 (Takara, Japan); GH43, the band of amplification; 5 is the band of xys-l5. (TIF) [file pone.0073729.s003.tif]

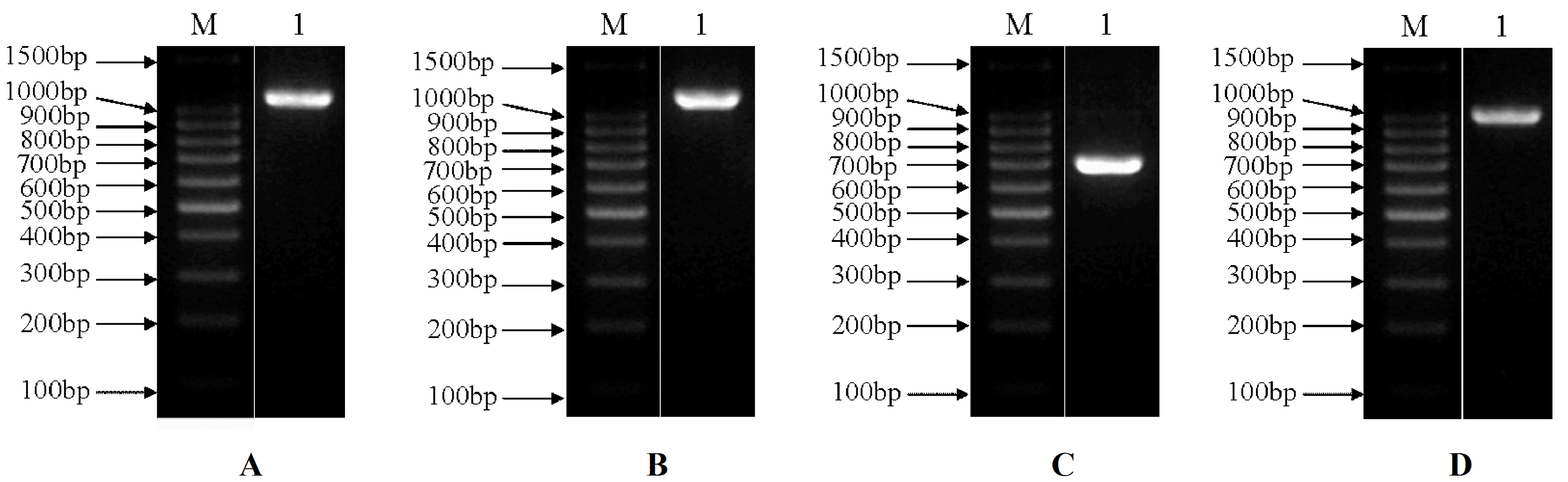

Supplement: Figure S4 — Electrophoretic gel images of PCR amplification of the CDS belonging to four novel xylanolytic enzyme genes. (A) PCR amplification of xyn-l1; M, 100 bp DNA Ladder (Real-Times, China); 1, the band of xyn-l1. (B) PCR amplification of xyn-l2; M, 100 bp DNA Ladder (Real-Times, China); 1, the band of xyn-l2. (C) PCR amplification of xyn-l4; M, 100 bp DNA Ladder (Real-Times, China); 1, the band of xyn-l4. (D) PCR amplification of xys-l5; M, 100 bp DNA Ladder (Real-Times, China); 1, the band of xys-l5. (TIF) [file pone.0073729.s004.tif]
